# M^3^G: Maximum Margin Microarray Gridding

**DOI:** 10.1186/1471-2105-11-49

**Published:** 2010-01-25

**Authors:** Dimitris Bariamis, Dimitris K Iakovidis, Dimitris Maroulis

**Affiliations:** 1 Department of Informatics and Telecommunications, University of Athens, Athens, Greece; 2 Department of Informatics and Computer Technology, Technological Educational Institute of Lamia, Lamia, Greece

## Abstract

**Background:**

Complementary DNA (cDNA) microarrays are a well established technology for studying gene expression. A microarray image is obtained by laser scanning a hybridized cDNA microarray, which consists of thousands of spots representing chains of cDNA sequences, arranged in a two-dimensional array. The separation of the spots into distinct cells is widely known as microarray image gridding.

**Methods:**

In this paper we propose M^3^G, a novel method for automatic gridding of cDNA microarray images based on the maximization of the margin between the rows and the columns of the spots. Initially the microarray image rotation is estimated and then a pre-processing algorithm is applied for a rough spot detection. In order to diminish the effect of artefacts, only a subset of the detected spots is selected by matching the distribution of the spot sizes to the normal distribution. Then, a set of grid lines is placed on the image in order to separate each pair of consecutive rows and columns of the selected spots. The optimal positioning of the lines is determined by maximizing the margin between these rows and columns by using a maximum margin linear classifier, effectively facilitating the localization of the spots.

**Results:**

The experimental evaluation was based on a reference set of microarray images containing more than two million spots in total. The results show that M^3^G outperforms state of the art methods, demonstrating robustness in the presence of noise and artefacts. More than 98% of the spots reside completely inside their respective grid cells, whereas the mean distance between the spot center and the grid cell center is 1.2 pixels.

**Conclusions:**

The proposed method performs highly accurate gridding in the presence of noise and artefacts, while taking into account the input image rotation. Thus, it provides the potential of achieving perfect gridding for the vast majority of the spots.

## Background

The process of protein synthesis inside the cells begins with the transcription of a gene sequence from DNA to messenger RNA (mRNA) in the cell nucleus. The mRNA is then transported outside the nucleus and the sequence encoded in the mRNA chain is translated into amino acids that form the corresponding protein for that particular sequence. Since proteins are translated directly from mRNA chains, the quantity of each mRNA chain that is present in a cell is indicative of the corresponding protein synthesis, i.e. the gene expression. The goal of a microarray experiment is the quantification of the amount of mRNA present in a test sample compared to that of a reference sample.

The first step of such an experiment is the isolation of the test and reference mRNA samples. These two samples are reverse-transcribed into complementary DNA (cDNA), amplified using polymerase chain reaction and labelled, usually by means of two distinct fluorescent dyes such as the red Cy5 and the green Cy3. The labelled cDNA is hybridized on a microarray device that consists of a solid substrate and a large number of spots, where single-stranded chains of known DNA sequences are attached. Each of these sequences corresponds to a part of a specific gene. The sample cDNA can only be hybridized with its complementary sequence. The hybridized microarray is then scanned at the wavelength of each dye and the output of the experiment is a high resolution greyscale digital image for each wavelength. Such an image consists of a matrix of blocks, each of which contains a number of rows and columns of spots. The grey level intensity of each spot signifies the degree of hybridization of the labelled cDNA sample to the known DNA sequences, thereby indicating the expression levels of the respective genes.

The gene expression levels are extracted from microarray images in three steps. The first step of this process is the separation of the blocks present in the image. The next step is gridding, i.e. the construction of a grid covering each block so that it isolates each spot into a distinct cell, enabling the localization of each spot. The last step involves the segmentation of the spots from the background of the image and the quantification of the intensity of each spot, which corresponds to the expression level of the respective gene.

The distance between the blocks of each image is significantly larger than the distance between the spots of each block, thus the blocks can easily be separated. A variety of approaches have been proposed for block separation and have achieved accurate results. These include the analysis of the distances between neighbouring spots [1] and the use of projections of the image pixels to the *x *and *y *axes [2,3]. In contrast to the block separation step, the process of gridding poses several challenges and has a significant impact on the accuracy of a microarray experiment [4]. A gridding algorithm should be able to grid images that include spots of various shapes, sizes and intensities, while being robust to noise and artefacts introduced at a microarray preparation stage, as well as rotation due to slight misalignments of the scanning robot coordinate system to the image coordinate system [5]. Furthermore, it is desirable that the gridding be automatically performed, without any user intervention that would possibly affect the microarray experiment, as well as limit the processing throughput of large amounts of microarray images.

Several methods have been proposed for microarray image gridding; they can be viewed in terms of automation as manual, semiautomated and fully automated [6]. However, most of the proposed methods are not fully automated and require manual tuning of parameters or other user intervention. For example, the state of the art method implemented in ImaGene [7] is semiautomated, requiring the tuning of a multitude of parameters, whereas in the manual gridding methods implemented in ScanAlyze [8] and SpotFinder [9], the process of gridding is performed interactively by the user. The method proposed by Brändle et al. [10] is parametric, requiring estimated values for several parameters.

Only a few state of the art methods have been proposed as providing automatic gridding, but most of them do not address all requirements of fully automatic gridding, i.e. handling of irregular spots and robustness against noise, artefacts and image rotation. The state of the art method proposed by Angulo et al. [11] is based on mathematical morphology and requires that grid rows and columns are strictly aligned with the *x *and *y *axes of the microarray image. The same requirement is imposed by the hill-climbing approach proposed by Rueda et al. [12]. A fully automatic region segmentation approach based on Markov random fields was proposed by Katzer et al. [13] but the results showed that its performance is diminishing in the presence of weakly expressed spots. The Bayesian grid matching method proposed by Hartelius et al. [14] employs an iterative algorithm to solve a complex deformable model for accurate microarray gridding, whereas methods producing simpler linear grids such as [13] and [15] have been proved highly accurate as well. Blekas et al. [16] proposed a method based on Gaussian mixture model, whereas later a methodology combining a stochastic search approach for the grid positioning and a Markov Chain Monte Carlo method was proposed by Antoniol et al. [17] to account for local deformations of the microarray image. However, this approach cannot be considered as fully automatic since it requires prior knowledge about the number of rows and columns of the spots in the microarray image. Another method based on Voronoi diagrams was proposed by Giannakeas et al. [3], however it requires that artificial spots are introduced in place of the spots that are very weakly expressed. Recently, a heuristic gridding approach based on a genetic algorithm was proposed by Zacharia et al. [15]. This algorithm provides a near optimal gridding outperforming the method proposed by Blekas et al. [16], while being robust to both noise and rotation. However, it is well known that the genetic optimization processes tend to require long processing times to converge, since a multitude of possible solutions has to be created and evaluated.

In this paper we propose a novel methodology for automatic cDNA microarray gridding based on a computationally efficient optimization approach. The proposed methodology is based on the maximization of the margin between the consecutive rows and columns of the microarray spots, which is implemented by training a linear maximum margin classifier with an automatically detected subset of spots on the microarray image. The classifier determines the optimal positioning of each grid line, whereas the use of the soft-margin variant provides robustness to outliers. This methodology, named M^3^G (Maximum Margin Microarray Gridding) is supported by a non-parametric Radon-based rotation estimator of general applicability for cDNA microarray images. The novel contributions of the proposed method include:

• Optimal grid line determination through margin maximization

• Fully automatic gridding without user intervention

• Robustness to a wide range of image imperfections, including rotation, artefacts, noise and weakly expressed spots

• Higher accuracy than the state of the art methods

All algorithms have been implemented under a GNU/Linux environment. The M^3^G software is publicly available [18] and also provided along with the manuscript [Additional file 1]. A preliminary version of the proposed methodology, which involved an arbitrary threshold setting in the spot detection process and a crude rotation estimator, has been presented in [19] along with a limited experimental evaluation.

## Methods

**Figure 1:**
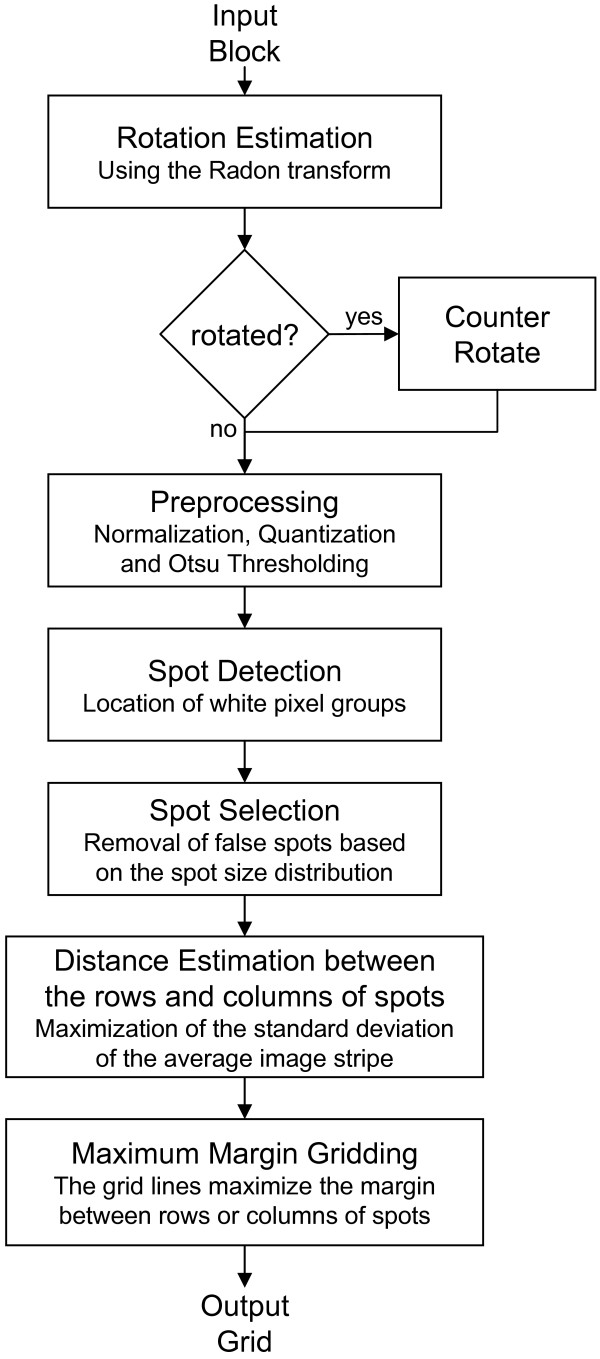
**Flowchart of the proposed method**.

The proposed method consists of the steps illustrated in the flowchart of Fig. 1.

### Rotation estimation

**Figure 2:**
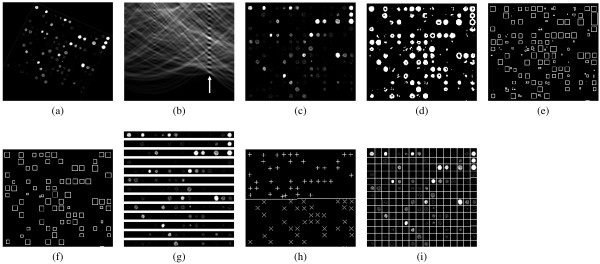
**Steps of the proposed method: (a) input microarray image, (b) result of the Radon transform (c) counter-rotated input image, (d) binarized image, (e) detected spots, (f) selected spots, (g) distance estimation between rows of spots, (h) determination of grid line and (i) gridded microarray image**.

The 16-bit greyscale microarray image (Fig. 2a) is initially analyzed by the Radon transform (Eq. 1), which is applied to estimate the image rotation angle. The Radon transform has been the method of choice for microarray rotation estimation in approaches such as [10].(1)

In the above equation, the grey level intensity of the image in pixel (*x, y*) is denoted by *I*(*x, y*). In the transformed image illustrated in Fig. 2b, the intensity of each pixel with coordinates (*a, r*) is equal to the integral of the image brightness over a straight line with an angle *a *to the *x *axis and a distance *r *to the origin. The rotation angle *θ *of the microarray image is estimated by locating the column with the highest mean brightness in the transformed image, which is denoted by the arrow. The image is subsequently counter-rotated by angle *θ *as illustrated by Fig. 2c.

### Preprocessing

The next step involves the pre-processing of the microarray image by linearly normalizing it so as to fit the intensity histogram to the full dynamic range of the 16-bit image. The image is then quantized to 256 grey levels in order to reduce the computational complexity of the next steps. Subsequently, the edges of the spots are detected by the application of the Sobel operator [20] on the normalized image. A threshold is determined automatically using the Otsu method [21] in order to binarize the image and isolate the sharpest edges that correspond to spots, as illustrated in Fig. 2d.

Since the image has been normalized to the full dynamic range and the result of the preprocessing step is a binarized image, the quantization error is expected to affect a very small number of pixels. Indeed, in the data set used for the evaluation of the proposed method (see Results), the percentage of the affected pixels in each binarized image was less than 0.01%, having no effect on the subsequent grid placement.

### Spot detection

The binarized image is then analyzed so as to locate all the groups of consecutive white pixels that reside on the same spot edge. Each of these groups is characterized by the location of the center pixel and the size of the group. Fig. 2e illustrates the detected groups by representing them as rectangles that circumscribe the pixels of each group. Ideally, each rectangle should contain the edge pixels of a single microarray spot, however depending on the threshold used, it might also include artefacts or multiple merged spots, due to the noise present in the image and the inter-spot proximity. A spot selection step is introduced in the next subsection, in order to refine the spots based on their shape and size characteristics resulting in the rectangles shown in Fig. 2f.

### Spot selection

The spot selection process aims to the removal of false spots introduced by noise and artefacts. In the spot selection step, the aspect ratios of the detected pixel groups are first evaluated. Considering that the ideal spot shape is circular, the rectangles (Fig. 2e) should not deviate much from being square, so that each rectangle contains only one microarray spot. Therefore, the aspect ratio of each spot must be close to unity. Then, a lower bound *s*_*min *_and an upper bound *s*_*max *_of the spot sizes are calculated so as to maximize the similarity of the spot size distribution to the normal distribution. The spots that have sizes out of the calculated bounds are considered false and discarded.

In order to quantify the similarity of the distribution of the spot sizes to the normal distribution, it has to be taken into account that the spot sizes can only be positive, in contrast to the normal distribution *N *(*x*; *μ*, *σ*) (Eq. 2) that also spans into the negatives. The comparison should therefore be made to a normal distribution for which the negative values are explicitly set to zero. Such a distribution *N*_*m*_(*x*; *μ*, *σ*) (Eq. 3) can be derived by nullifying the probability of *N *(*x*; *μ*, *σ*) for *x *< 0 and scaling it accordingly so that the total probability remains equal to unity. The corresponding cumulative distributions *C*(*x*; *μ*, *σ*) and *C*_*m*_(*x*; *μ*, *σ*) are expressed by Eqs. 4 and 5 respectively.(2)

A measure of dissimilarity *E *between the discrete probability distribution of the spot sizes and the continuous probability distribution *N*_*m*_(*x*; *μ*, *σ*) can be established based on their respective cumulative distribution functions. The cumulative histogram of the spot sizes *C*_*h*_(*x*) is defined as a function of the histogram *h*(*x*) as shown in Eq. 6. The dissimilarity *E *is defined as the total area between *C*_*h*_(*x*) and *C*_*m*_(*x*; *μ*, *σ*) as shown in Eq. 7.(6)

**Figure 3:**
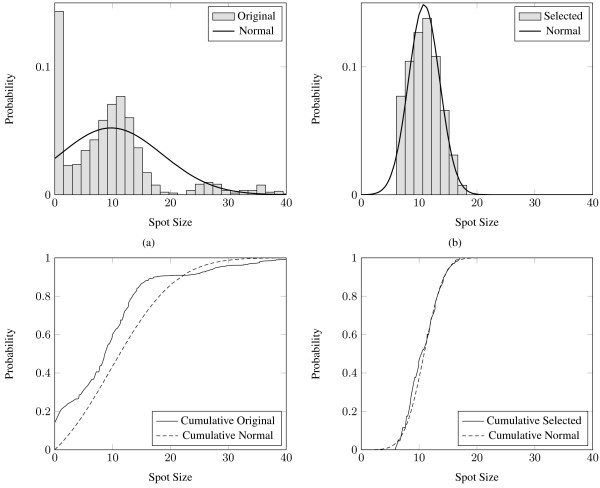
**The (a) original, (b) selected, (c) cumulative original and (d) cumulative selected spot size distributions compared to their respective modified normal distributions**.

The optimal bounds *s*_*min *_and *s*_*max *_are calculated so as to minimize the dissimilarity *E *defined above. By selecting the spots with sizes within the range defined by these bounds, the resulting cumulative spot size distribution closely resembles the normal distribution, as illustrated in the example of Fig. 3. In this case, any spot that is smaller than *s*_*min *_= 6.4 pixels or larger than *s*_*max *_= 17.1 pixels is considered false and discarded. It is evident that the cumulative histogram of the selected spots almost coincides with the cumulative normal distribution (Fig. 3d), whereas the original cumulative distribution (Fig. 3c) differs substantially from the respective cumulative normal distribution.

### Distance estimation between consecutive rows and columns

**Figure 4:**
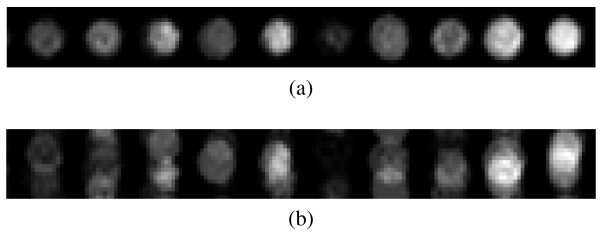
**Detail of average stripes for the horizontal direction and for (a) optimal *d*_*r *_and (b) suboptimal *d*_*r*_**.

The optimal distance between spot rows is calculated by segmenting the input microarray image into horizontal stripes with a height of *d*_*r *_pixels, as shown in Fig. 2g, which are then averaged. If *d*_*r *_is selected so that it is equal to the distance between the rows, the spots of all rows will be in the same relative positions in the horizontal stripes, therefore they will be highly overlapping in the resulting average stripe. Thus, the average stripe will contain well defined spot areas, as illustrated in Fig. 4a. If a suboptimal value of *d*_*r *_is selected, the spots will reside in different relative positions in the horizontal stripes and will thus blend with the background in the average stripe (Fig. 4b). The optimal value of *d*_*r *_is selected by maximizing the standard deviation of the pixel intensities of the average stripe. The standard deviation can be used as an effective measure of spot overlap, since high values of the standard deviation indicate distinct dark and bright areas, whereas low values of the standard deviation indicate abundant grey areas. Thus, the standard deviation should be maximized with respect to *d*_*r *_in order to obtain the optimal value of *d*_*r*_. The optimal column width *d*_*c *_is likewise estimated using vertical stripes.

A wide range of *d*_*r *_values is tested in order to find the optimal value, ensuring successful estimation without any user intervention. The standard deviation  of the average stripes is calculated for all values of *d*_*r *_within that range, using a small real valued step. From all the tested values of *d*_*r*_, those that result in local maxima of the standard deviation are selected. These local maxima are most often located on multiples of the optimal *d*_*r*_, since such an estimation also results in highly overlapping spots. For each of the selected *d*_*r *_values, the mean of the resulting standard deviation  in its neighbourhood is calculated. The neighbourhood for the calculation of each  is equal to the range between its adjacent local maxima. The value of *d*_*r *_that results in the highest value of the  ratio is selected as optimal.

Another method for estimating the distances between rows and columns of spots has been proposed by Ceccarelli et al. [22]. It employs the Orientation Matching (OM) and Radon transforms in order to extract the spot positions and grid rotation respectively. Subsequently, the spot locations are projected on the axes of the grid and the distance between rows and columns is estimated. This method requires prior knowledge about the radii of the spots and uses a filter for noise reduction. In contrast to this approach, the proposed method performs distance estimation without any parameters or arbitrarily selected filters, based on the maximization of the standard deviation of the average stripe. This maximization over a wide range of *d*_*r *_values allows successful estimation without any user-defined parameters, whereas the use of the average stripe acts as a low pass filter, allowing high tolerance to noise. An evaluation of the distance estimation for noisy images has been included in the Results section.

### Maximum margin gridding

In order to determine the optimal grid lines, each spot that has been selected by the spot selection step (Fig. 2e) is represented by a vector , *i *= 1... *N*, where *N *is the total number of selected spots in the image and the components of each vector  are the coordinates of the spot centre. These vectors are assigned into distinct rows and columns, based on the distances *d*_*r *_and *d*_*c*_, which were previously estimated. Each pair of consecutive rows or columns of spots can now be separated by a single separating line. The optimal separating line is positioned so as to maximize the margin between the rows or columns of the spots. For a pair of rows numbered *k *and *k *+ 1, the vectors that belong to row *k *or to any row above it are assigned a class label *c*_*i *_= +1 and the vectors that belong to row *k *+ 1 or to any row below it are assigned a class label *c*_*i *_= -1. These vectors , along with their respective class labels *c*_*i *_are provided as a training set to a linear Support Vector Machine (SVM) classifier [23], which produces the maximum margin grid line.

In this particular application, the SVM classifier is provided with the aforementioned training set . By solving a quadratic programming optimization problem, it produces the normal vector  and the parameter *b *of the separating line , which maximizes the margin between vectors  of different classes, i.e. the margin between spots of distinct rows or columns. The width of the margin is equal to 2/||*w*||, therefore the widest margin is found by minimizing ||*w*|| under the constraints *c*_*i*_( - *b*) ≥ 1, i.e. requiring that all the spots lie on the correct side of the resulting grid line.

The support vector machine described above is called a *hard-margin *SVM and does not take into account any outliers. One of its properties is that the separating line is solely determined by the vectors that that lie on the edges of the margin, called *support vectors*. In a linear SVM, a very small number of support vectors determine the separating line and the margin. In the case of outliers present inside the margin, the positioning of the separating line will be exclusively determined by the outliers and will thus be suboptimal for gridding. This problem can be solved using the *soft-margin *SVM, where a slack variable *ξ*_*i *_is introduced for each vector . The constraints are then formulated as  and the separating hyperplane can be found by minimizing(8)

where *C *is a cost parameter that determines the effect of outliers on the positioning of the resulting grid line. Large values of *C *result in a grid line that is mostly determined by any outliers, while on the other hand, smaller values of *C *result in a grid line that follows the general trend of the spot locations given to the classifier, virtually ignoring any outliers. The hard-margin classifier is equivalent to a soft-margin classifier with an infinitely large *C *[24].

The soft-margin SVM is employed, in order to diminish the effects of misdetected spots that result from artefacts or noise. A small fraction of these outliers might have a shape and size similar to valid spots and could therefore pass through the selection step without being discarded. The soft-margin SVM ensures that such outliers will not have an impact on the produced grid lines. For ideal microarray images, where all spots could be successfully detected and no outliers are present, a hard-margin SVM could be used as well, but a gridding application for real microarray images requires robustness against outliers. Furthermore, in ideal noiseless images, the training set for the SVM classifier would consist only of the necessary spots, i.e. those residing on rows *k *and *k *+ 1. However, in real microarray images, there are cases where several consecutive spots might be very weakly expressed and therefore not detected. In order to cope with this problem, spots from rows above *k *and below *k *+ 1 are included in the training set, providing redundant data to the classifier to ensure successful gridding. Using an algorithm based on the Sequential Minimal Optimization (SMO) to solve the SVM optimization problem [25], the additional data introduces only a small computational overhead, since such algorithms evaluate vectors that are far from the separating line in only the first few iterations of their outer loops [26,27]. The SVM has been selected over similar methods for the determination of the grid lines, such as a least squares fit, because the soft margin SVM is adjustable with regards to its tolerance to outliers through the cost parameter *C *[28].

In the case that row *k *contains less than two detected spots, the two grid lines that separate this row from rows *k *- 1 and *k *+ 1 cannot be determined by the use of the SVM classifier. This is a rather rare case considering that the image is normalized during the preprocessing step. To cope with this limitation, the endpoints of the two grid lines are positioned equidistantly between the endpoints of the first neighboring grid lines above and below them. In the case where the top or bottom rows of spots contain less than two spots, the endpoints of the grid lines that cannot be determined are positioned *d*_*r *_pixels further from the nearest grid lines.

**Figure 5:**
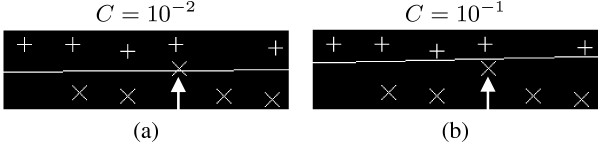
**The effect of an outlier as a function of the SVM cost parameter *C***. (a) Small value of *C*, (b) Large value of *C*.

Fig. 5 illustrates the case of gridding in the presence of an obvious outlier, denoted by the arrow. It is evident that for a small value for the cost parameter C (Fig. 5a) the margin is determined by the other spots in the row and the outlier is ignored, whereas for larger values of C (Fig. 5b) the outlier affects the positioning of the separating line by moving it significantly closer to the vectors of the top row, reducing the margin and rendering it suboptimal for gridding.

## Results and Discussion

### Data sets and evaluation method

**Figure 6:**
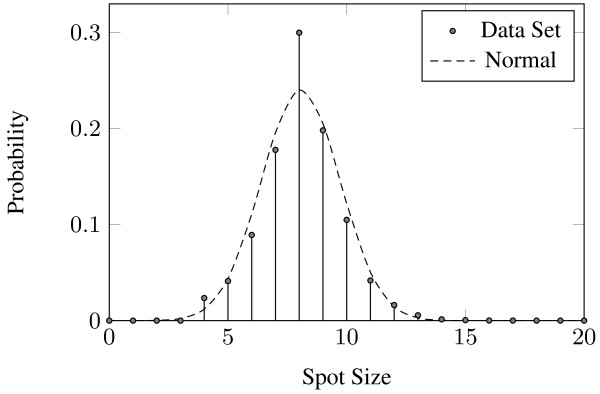
**The distribution of spot diameters in the data set compared to the modified normal distribution *N*_*m *_(*x *; *μ*, *σ*) with *μ *= 8.07 and *σ *= 1.66**.

Two data sets were used for the evaluation of M^3^G. The first data set consists of 54 cDNA microarray images from the Stanford Microarray Database [29]. The images are TIFF files with a resolution of 1900 × 5500 pixels and 16-bit grey level depth. Each image includes 48 blocks of 870 spots each, resulting in a total of 2,255,040 spots in the data set. These images have been produced for the study of the gene expression profiles of 54 specimens of BCR-ABL-positive and -negative acute lymphoblastic leukemia [30]. This data set is a superset of the one used by the *preliminary version *of the proposed method [19] and the *genetic algorithm *approach proposed in [15]. This data set is accompanied by ground truth annotations regarding the positions and the sizes of the spots. Fig. 6 visually validates the resemblance of the distribution of the sizes of the spots in the data set to the *N*_*m*_(*x*; *μ*, *σ*) distribution.

The second data set consists of 10 microarray blocks selected from distinct microarray images that were artificially created or obtained from public microarray databases. The blocks are stored in TIFF files with 16-bit grey level depth. This data set has been used for the evaluation of the method proposed by Blekas et al. [16] and has been obtained from the authors of [16].

In order to produce directly comparable results with the aforementioned methods, the same statistical analysis is performed. Each spot was evaluated as being perfectly, marginally or incorrectly gridded when the percentage of its pixels contained within its grid cell is 100%, more than 80%, or less than 80% respectively.

### Comparative evaluation

The evaluation results of M^3^G for the first data set are shown in Table 1, along with the corresponding results of the *preliminary version *of the proposed method and the *genetic algorithm *approach. In order to evaluate the sensitivity of the proposed method to missing spots, two experiments were conducted: in the first, all selected spots were used for the SVM training process, whereas in the second, half of the selected spots were randomly discarded. Both experiments resulted in perfect gridding for more than 98% of the spots in the data set, whereas only up to 1.6% and 0.3% of the spots were marginally and incorrectly gridded respectively. These results illustrate that the proposed method can achieve almost perfect gridding, even in the case of significantly fewer detected spots.

**Table 1:** Comparison of gridding results for the first data set

	Perfect	Marginal	Incorrect
Zacharia et al. [15]	94.6	4.8	0.6
Preliminary method [19]	95.1	4.5	0.4
M^3^G (all spots)	98.3	1.5	0.2
M^3^G (50% of spots)	98.1	1.6	0.3

The evaluation results for the second data set are shown in Table 2 and reveal that the proposed method achieves a significantly lower percentage of marginally and incorrectly gridded spots compared to the user-guided approaches [8,9] and the automatic approaches [15,16].

**Table 2:** Comparison of gridding results for the second data set

	Perfect	Marginal	Incorrect
ScanAlyze [8]	48.7	22.6	28.7
SpotFinder [9]	72.8	14.3	12.9
Zacharia et al. [15]	94.4	5.1	0.5
Blekas et al. [16]	89.6	9.2	1.2
M^3^G	98.0	1.7	0.3

In comparison to the preliminary version [19], several improvements allow a significant enhancement of gridding accuracy:

• Automatic selection of spots based on the distribution of their sizes

• Automatic determination of the operating parameters, such as the edge detection threshold and the upper and lower bounds of the sizes of the spots

• Inclusion of all selected spots into the training set of each SVM classifier instead of only pairs of rows or columns of selected spots

• More accurate rotation detection using the Radon transform

The method used for the evaluation of the gridding accuracy by [3] involves the measurement of the distance between each spot center and the center of its respective grid cell. The mean value and standard deviation of these distances are used to evaluate the localization of the spots by the grid. The results of M^3^G as compared to the ones presented in [3] are shown in Table 3. The proposed method achieves a 50% smaller mean distance and a 33% smaller standard deviation as compared to the best results presented in [3], illustrating the advantageous performance of the proposed method with regards to the localization of the spots. Even when half of the selected spots are randomly discarded to simulate the case of significantly fewer detected spots, M^3^G still achieves a better localization than the best results of [3].

**Table 3:** Euclidean distances between spot centers and grid cell centers

	Mean	Standard Deviation
Giannakeas et al. (Red)	2.73 pixels	1.10 pixels
Giannakeas et al. (Green)	2.34 pixels	1.07 pixels
M^3^G (all spots)	1.17 pixels	0.71 pixels
M^3^G (50% of spots)	1.21 pixels	1.04 pixels

**Figure 7:**
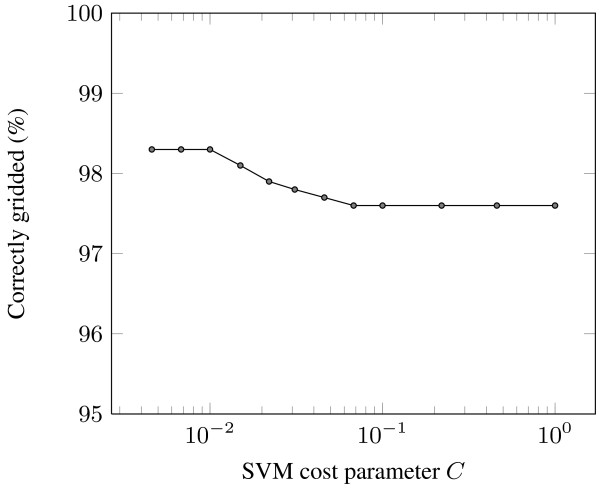
**Percentage of correctly gridded spots as a function of the SVM cost parameter *C***.

The gridding performance of M^3^G was evaluated by following a grid search approach for the determination of the optimal value for the SVM cost parameter *C*. The results are presented in Fig. 7. The SVM cost parameter *C *determines the effect that outliers or noise might have on the positioning of the separating lines produced by the SVM, therefore a small value of *C *should be selected for successful gridding. The choice of *C *= 10^-2 ^is supported by the results, as it produces the most accurately gridded spots compared to the other values of *C*. Lower values of *C *do not increase the achieved accuracy, but the choice of a larger value results in a reduction of the achieved accuracy due to the corresponding increase of significance of outliers.

**Figure 8:**
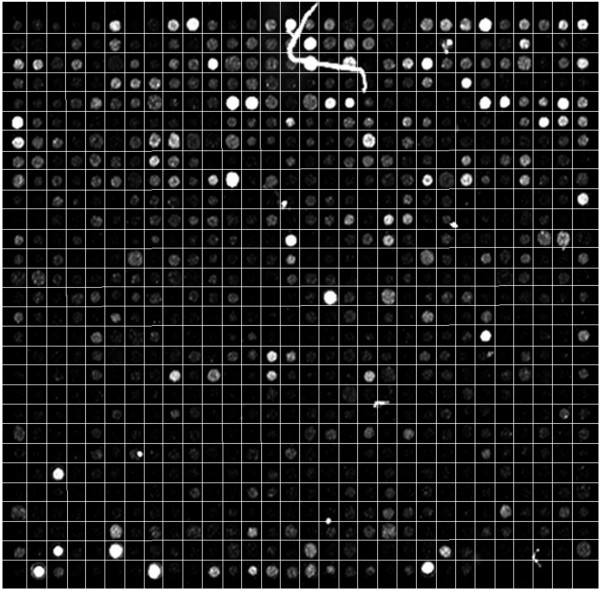
**Example of successful gridding in the presence of a large and bright artefact**.

**Figure 9:**
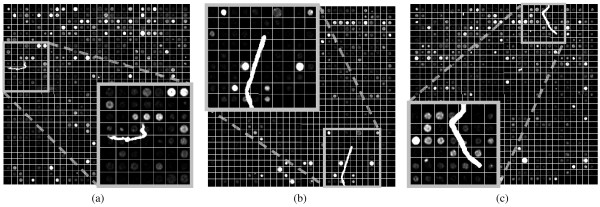
**Details of successful gridding for microarray images with bright artefacts**.

Fig. 8 illustrates the gridding that results from the application of M^3^G on a microarray image area that includes a large and bright artefact. Even in the vicinity of the artefact, the gridding is not affected by its presence. Fig. 9 illustrates the resulting gridding for three more such images, including a detailed view of the area around each artefact. Despite the presence of these artefacts, the proposed method achieves successful gridding in all those cases.

### Time performance

The proposed method was also evaluated with regards to its computational time requirements. The evaluation platform is based on an Athlon 64 X2 3800+ processor and includes 3GB of RAM, whereas the blocks used belong to the first data set and their dimensions are roughly 450 × 450 pixels. For the first block in each microarray image, our method requires 18 seconds of processing time, most of which is used for estimating the distance between consecutive rows and columns of spots. For any subsequent blocks of the same image, the range of possible values for *d*_*r *_and *d*_*c *_is reduced, resulting in 10 seconds of processing time. It is worth noting that the processing times mentioned above can be significantly reduced by optimizing the implementation and by using multiple cores of the processor, as the most time consuming parts of the gridding process can be efficiently parallelized. The genetic algorithm approach [15] requires a processing time of 92 seconds for each microarray image block, which is nearly one order of magnitude larger than the time required by M^3^G.

### Evaluation of the rotation detection

In order to assess the performance of the rotation estimation step, the images in the first data set were randomly rotated by angle *θ*_*real *_ranging from -25° to +25°. The proposed rotation detection method was then used to compute an estimate *θ*_*est *_of the rotation for each image. Based on that estimate, the images were counter-rotated and subsequently gridded. The evaluation was performed based on the mean and standard deviation of the difference between the real and estimated rotation of the images Δ*θ *= *θ*_*real *_- *θ*_*est*_. Both the mean difference *μ*_Δ*θ *_and the standard deviation *σ*_Δ*θ *_were below 0.1°. The accuracy achieved when gridding the counter-rotated images was within 0.3% of the accuracy obtained using the original images, thus the introduction of rotation results in only negligible variation of the gridding accuracy.

### Evaluation of the distance estimation

The distance estimation step was evaluated with regards to its tolerance to noise. Additive Gaussian noise was introduced to fifty randomly selected blocks from distinct real microarray images of the first data set. The standard deviations used were *σ *= 250, 500 and 1000, resulting in mean signal to noise ratios of 9 dB, 5.5 dB and 1 dB respectively, whereas in several cases the introduced noise was stronger than the signal in the image. For all the images and noise levels tested, the distance estimation step displayed a negligible variance of up to 0.02 pixels, illustrating that the use of the average stripe for distance estimation provides very high tolerance to noise.

## Conclusions

In this paper we presented M^3^G, a novel method for gridding cDNA microarray images without user intervention, based on the maximization of the margin between consecutive rows and columns of spots. The proposed method involves several preprocessing steps, including a Radon-based rotation estimation for the microarray image, as well as spot detection and selection. The distance between rows and columns of spots is then estimated and the positions of the selected spots are used to train a set of linear soft-margin Support Vector Machine classifiers. The use of soft-margin SVMs allows high tolerance to outliers that result from artefacts and noise, whereas the use of redundant vectors in the SVM training set and the automatic determination of the operating parameters facilitate a substantial increase in gridding accuracy. Overall, the proposed method achieves successful automatic gridding of cDNA microarray images in the presence of irregular spots, noise and artefacts, as well as image rotation.

The experimental results on reference DNA microarray images containing more than two million spots showed that the proposed method outperforms the most accurate state of the art methods, providing the potential of achieving perfect gridding for the vast majority of the spots.

## Competing interests

The authors declare that they have no competing interests.

## Authors' contributions

All authors were equally involved in the conception and design of the M^3^G method, as well as the writing and revision of the manuscript. DB developed the implementation of M^3^G and performed the experiments.

## Supplementary Material

Additional file 1 **M^3^G Software**. All algorithms have been implemented under a GNU/Linux environment. The M^3^G software is publicly available at the Downloads page of http://rtsimage.di.uoa.gr/ and also provided along with the manuscript as an additional file. Click here for file
