# Supplementary figures and images for: M3G: Maximum Margin Microarray Gridding
Source: BMC Bioinformatics. 2010 Jan 25;11:49. doi: 10.1186/1471-2105-11-49 (PMC2823709; doi:10.1186/1471-2105-11-49)

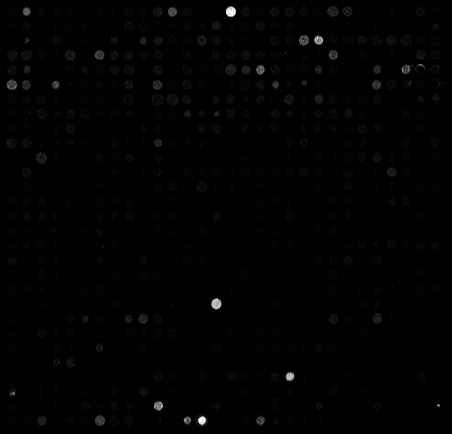

Supplement: Additional file 1 — M3G Software. All algorithms have been implemented under a GNU/Linux environment. The M3G software is publicly available at the Downloads page of http://rtsimage.di.uoa.gr/ and also provided along with the manuscript as an additional file. [file 1471-2105-11-49-S1.TGZ › m3g-0.01/images/64309_ch1-01.tif]

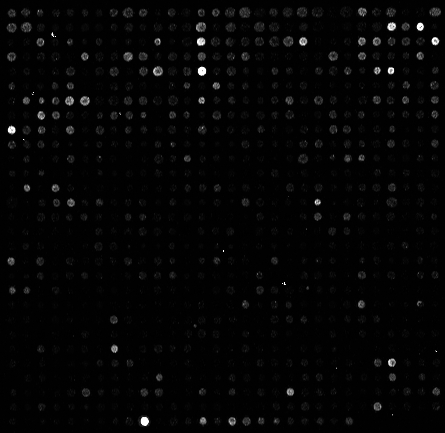

Supplement: Additional file 1 — M3G Software. All algorithms have been implemented under a GNU/Linux environment. The M3G software is publicly available at the Downloads page of http://rtsimage.di.uoa.gr/ and also provided along with the manuscript as an additional file. [file 1471-2105-11-49-S1.TGZ › m3g-0.01/images/64358_ch1-31.tif]

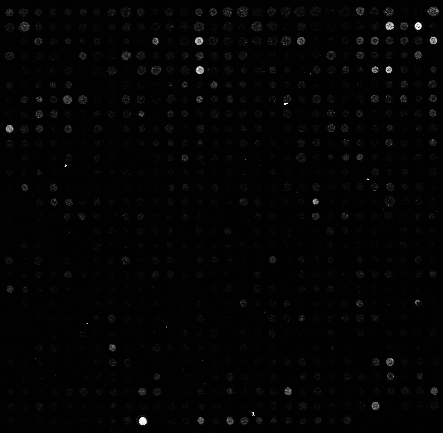

Supplement: Additional file 1 — M3G Software. All algorithms have been implemented under a GNU/Linux environment. The M3G software is publicly available at the Downloads page of http://rtsimage.di.uoa.gr/ and also provided along with the manuscript as an additional file. [file 1471-2105-11-49-S1.TGZ › m3g-0.01/images/64318_ch1-31.tif]

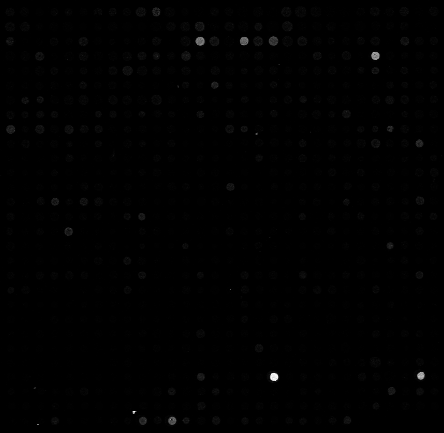

Supplement: Additional file 1 — M3G Software. All algorithms have been implemented under a GNU/Linux environment. The M3G software is publicly available at the Downloads page of http://rtsimage.di.uoa.gr/ and also provided along with the manuscript as an additional file. [file 1471-2105-11-49-S1.TGZ › m3g-0.01/images/64342_ch1-39.tif]

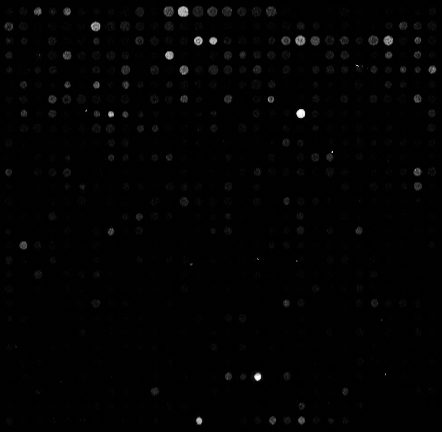

Supplement: Additional file 1 — M3G Software. All algorithms have been implemented under a GNU/Linux environment. The M3G software is publicly available at the Downloads page of http://rtsimage.di.uoa.gr/ and also provided along with the manuscript as an additional file. [file 1471-2105-11-49-S1.TGZ › m3g-0.01/images/64350_ch1-27.tif]

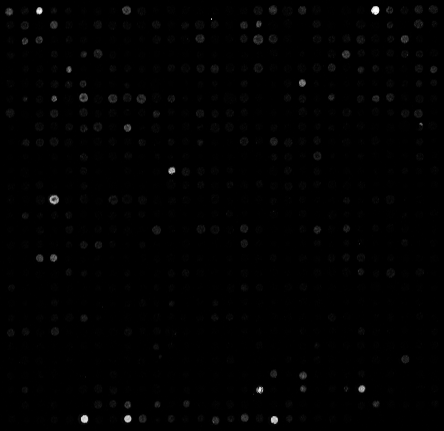

Supplement: Additional file 1 — M3G Software. All algorithms have been implemented under a GNU/Linux environment. The M3G software is publicly available at the Downloads page of http://rtsimage.di.uoa.gr/ and also provided along with the manuscript as an additional file. [file 1471-2105-11-49-S1.TGZ › m3g-0.01/images/64336_ch1-40.tif]

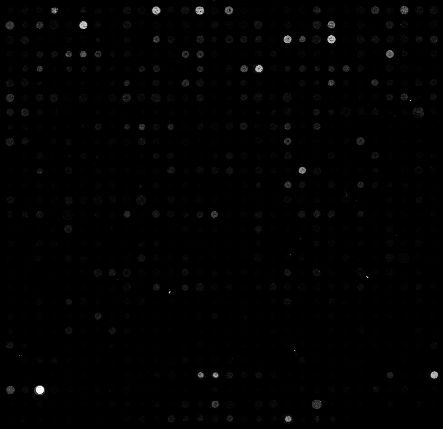

Supplement: Additional file 1 — M3G Software. All algorithms have been implemented under a GNU/Linux environment. The M3G software is publicly available at the Downloads page of http://rtsimage.di.uoa.gr/ and also provided along with the manuscript as an additional file. [file 1471-2105-11-49-S1.TGZ › m3g-0.01/images/64361_ch1-26.tif]

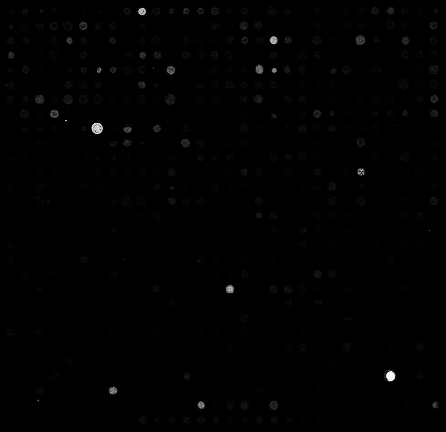

Supplement: Additional file 1 — M3G Software. All algorithms have been implemented under a GNU/Linux environment. The M3G software is publicly available at the Downloads page of http://rtsimage.di.uoa.gr/ and also provided along with the manuscript as an additional file. [file 1471-2105-11-49-S1.TGZ › m3g-0.01/images/64346_ch1-20.tif]

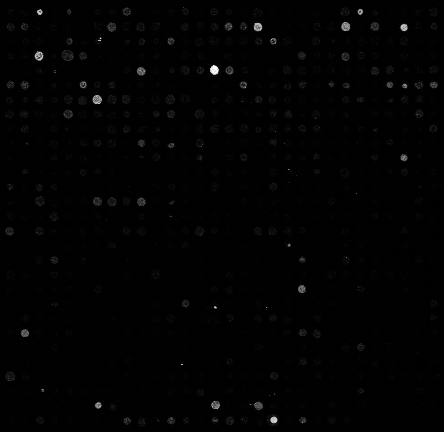

Supplement: Additional file 1 — M3G Software. All algorithms have been implemented under a GNU/Linux environment. The M3G software is publicly available at the Downloads page of http://rtsimage.di.uoa.gr/ and also provided along with the manuscript as an additional file. [file 1471-2105-11-49-S1.TGZ › m3g-0.01/images/64346_ch1-24.tif]

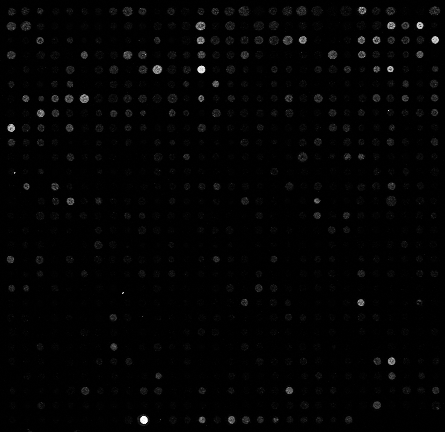

Supplement: Additional file 1 — M3G Software. All algorithms have been implemented under a GNU/Linux environment. The M3G software is publicly available at the Downloads page of http://rtsimage.di.uoa.gr/ and also provided along with the manuscript as an additional file. [file 1471-2105-11-49-S1.TGZ › m3g-0.01/images/64354_ch1-31.tif]

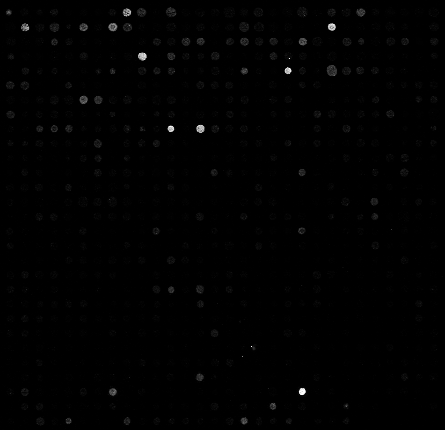

Supplement: Additional file 1 — M3G Software. All algorithms have been implemented under a GNU/Linux environment. The M3G software is publicly available at the Downloads page of http://rtsimage.di.uoa.gr/ and also provided along with the manuscript as an additional file. [file 1471-2105-11-49-S1.TGZ › m3g-0.01/images/64314_ch1-44.tif]

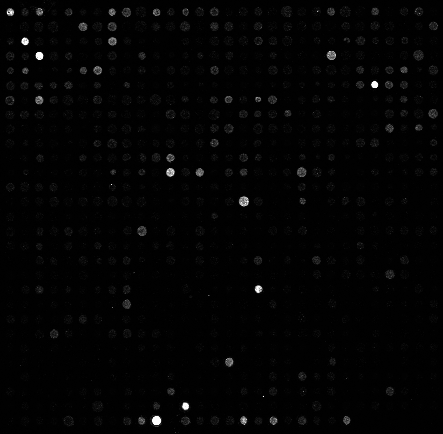

Supplement: Additional file 1 — M3G Software. All algorithms have been implemented under a GNU/Linux environment. The M3G software is publicly available at the Downloads page of http://rtsimage.di.uoa.gr/ and also provided along with the manuscript as an additional file. [file 1471-2105-11-49-S1.TGZ › m3g-0.01/images/64315_ch1-06.tif]

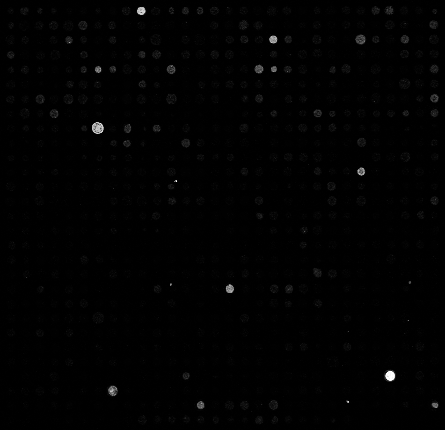

Supplement: Additional file 1 — M3G Software. All algorithms have been implemented under a GNU/Linux environment. The M3G software is publicly available at the Downloads page of http://rtsimage.di.uoa.gr/ and also provided along with the manuscript as an additional file. [file 1471-2105-11-49-S1.TGZ › m3g-0.01/images/64315_ch1-20.tif]

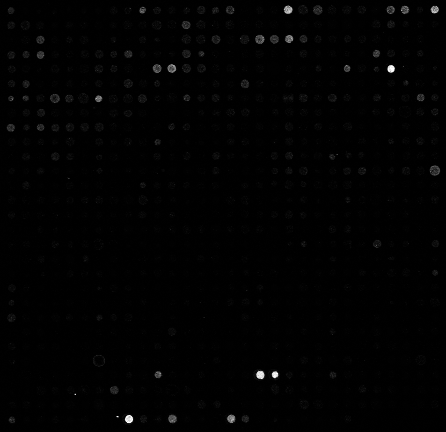

Supplement: Additional file 1 — M3G Software. All algorithms have been implemented under a GNU/Linux environment. The M3G software is publicly available at the Downloads page of http://rtsimage.di.uoa.gr/ and also provided along with the manuscript as an additional file. [file 1471-2105-11-49-S1.TGZ › m3g-0.01/images/64315_ch1-21.tif]

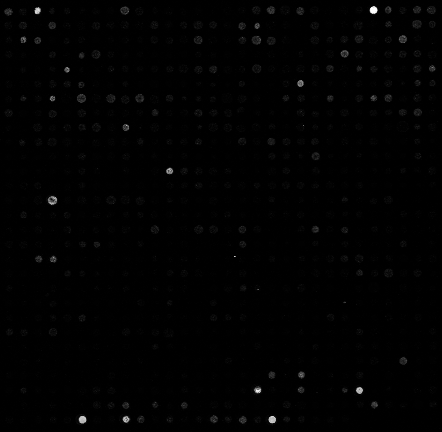

Supplement: Additional file 1 — M3G Software. All algorithms have been implemented under a GNU/Linux environment. The M3G software is publicly available at the Downloads page of http://rtsimage.di.uoa.gr/ and also provided along with the manuscript as an additional file. [file 1471-2105-11-49-S1.TGZ › m3g-0.01/images/64315_ch1-40.tif]
